# Supplementary material for: The spatial and temporal variation of fine particulate matter pollution in Ethiopia: Data from the Atmospheric Composition Analysis Group (1998–2019)
Source: PLoS One. 2023 Mar 24;18(3):e0283457. doi: 10.1371/journal.pone.0283457 (PMC10038283; doi:10.1371/journal.pone.0283457)
Supplement: S1 Table — (DOCX) [file pone.0283457.s004.docx]

| **Regions** | **1998** | **1999** | **2000** | **2001** | **2002** | **2003** | **2004** | **2005** | **2006** | **2007** | **2008** | **2009** | **2010** | **2011** | **2012** | **2013** | **2014** | **2015** | **2016** | **2017** | **2018** | **2019** |
| --- | --- | --- | --- | --- | --- | --- | --- | --- | --- | --- | --- | --- | --- | --- | --- | --- | --- | --- | --- | --- | --- | --- |
| Addis Ababa | 15.5 | 14.1 | 16.0 | 21.0 | 19.4 | 17.0 | 17.0 | 17.4 | 19.4 | 18.6 | 20.9 | 20.1 | 23.1 | 24.4 | 23.7 | 24.2 | 20.9 | 23.5 | 23.5 | 22.0 | 22.4 | 22.8 |
| Afar | 22.7 | 24.1 | 27.0 | 24.6 | 25.9 | 24.5 | 24.2 | 25.5 | 25.2 | 24.7 | 29.4 | 28.8 | 29.3 | 31.3 | 35.8 | 33.7 | 26.6 | 32.0 | 28.5 | 29.7 | 29.7 | 30.9 |
| Amhara | 16.6 | 15.5 | 17.5 | 20.7 | 20.2 | 19.6 | 19.2 | 20.2 | 20.6 | 20.2 | 20.5 | 21.4 | 23.5 | 24.9 | 24.0 | 23.7 | 19.9 | 24.2 | 22.5 | 24.1 | 23.3 | 25.0 |
| Benishangul-Gumuz | 19.0 | 14.2 | 16.6 | 20.7 | 20.5 | 19.2 | 18.9 | 20.2 | 19.6 | 20.0 | 18.9 | 20.2 | 22.1 | 23.8 | 22.9 | 21.9 | 17.7 | 23.4 | 21.0 | 23.0 | 21.6 | 23.2 |
| Dire Dawa | 11.0 | 11.3 | 13.1 | 16.7 | 16.7 | 14.2 | 13.8 | 14.1 | 14.6 | 15.6 | 19.0 | 18.7 | 18.0 | 20.5 | 21.6 | 19.6 | 17.0 | 21.1 | 18.1 | 18.9 | 17.9 | 18.1 |
| Gambela Peoples | 24.6 | 18.4 | 20.4 | 18.5 | 19.9 | 18.0 | 18.0 | 19.1 | 19.0 | 21.2 | 19.9 | 19.7 | 20.3 | 23.0 | 23.2 | 21.7 | 18.4 | 24.9 | 22.0 | 23.0 | 23.1 | 23.2 |
| Harari People | 10.1 | 10.3 | 11.6 | 15.8 | 15.1 | 12.1 | 11.7 | 11.9 | 12.6 | 13.0 | 15.3 | 15.9 | 15.7 | 17.9 | 18.7 | 17.6 | 15.9 | 17.7 | 16.9 | 15.8 | 14.9 | 14.4 |
| Oromia | 13.0 | 11.0 | 12.4 | 16.5 | 15.5 | 12.6 | 12.2 | 12.8 | 13.4 | 13.9 | 15.1 | 15.8 | 16.6 | 18.5 | 18.4 | 18.2 | 16.1 | 17.9 | 17.5 | 16.2 | 16.0 | 15.9 |
| Somali | 11.6 | 12.5 | 13.2 | 13.1 | 13.2 | 10.8 | 9.7 | 10.2 | 10.0 | 10.3 | 12.8 | 13.0 | 12.2 | 13.4 | 14.8 | 13.9 | 12.3 | 12.9 | 12.5 | 12.8 | 12.1 | 12.1 |
| SNNP | 15.2 | 11.3 | 13.0 | 16.1 | 15.2 | 13.6 | 12.9 | 13.2 | 14.0 | 15.2 | 16.4 | 15.0 | 16.5 | 19.0 | 18.6 | 18.3 | 16.8 | 18.2 | 17.7 | 16.9 | 17.4 | 17.0 |
| Tigray | 20.9 | 19.6 | 22.4 | 20.4 | 20.0 | 19.8 | 18.4 | 19.7 | 18.8 | 19.3 | 19.6 | 22.5 | 23.2 | 23.9 | 24.0 | 24.3 | 19.2 | 23.6 | 22.2 | 22.6 | 22.3 | 23.9 |
| Ethiopia (Country average) | 15.1 | 13.9 | 15.5 | 17.2 | 16.9 | 15 | 14.3 | 15.1 | 15.2 | 15.6 | 17.1 | 17.4 | 18 | 19.7 | 20.2 | 19.6 | 16.8 | 19.3 | 18.2 | 18.3 | 18 | 18.4 |

Regional level annual mean PM_2.5_ in μgm^-3^ in table
