# Supplementary material for: The spatial and temporal variation of fine particulate matter pollution in Ethiopia: Data from the Atmospheric Composition Analysis Group (1998–2019)
Source: PLoS One. 2023 Mar 24;18(3):e0283457. doi: 10.1371/journal.pone.0283457 (PMC10038283; doi:10.1371/journal.pone.0283457)
Supplement: Attachment — Submitted filename: Resposne to Reviewers.docx [file pone.0283457.s005.docx]

Primarily, I would like to thank the PlosOne journal and the reviewers for their suggestions and comments on the manuscript.

| **Reviewer 1 comments** |  |  |
| --- | --- | --- |
| **Reviewer comment** | **Authors response** | **Line number** |
| Although the analysis presented is sound, the description is not detailed enough. There needs to be a more thorough description of the results and their implications as well as a detailed background and context in the results section. Therefore, I recommend major revision before this manuscript can be accepted for publication in PLOS ONE. | Thank you, for the comment we have made appropriate improvement in the results section. We have added the results of the clustering and outlier analysis with the data |  |
| 1. Results section: This section is too limited. It needs more details to add context to the results. For example, what does "significant spatial dependency" mean? What does it mean to say the PM concentrations are distributed non-random, clustered or dispersed. These results should be quantified and explained in more detail. | We accepted the comment made necessary changes | 275, 277-279, 283-286, 290-291, 293-299 |
| 2. Strengths and limitations section: Even though the satellite derived PM2.5 product has not been validated in the study region due to the lack of ground observations, it must have undergone validation by measurements in other locations. What are the conclusions of those validations. The authors need to discuss the general uncertainty in this product using literature review. | We accepted the comment and discussed the results of these validations | 363-369 |
| 3. The manuscript will improve with proof-reading and correcting grammatical and language errors. | We accepted the comment, gone through the manuscript and made changes |  |
| Line 28: This sentence is missing a verb. You might add "are" after "strong policies" | Thank you, we made the change | 28 |
| Line 30: It would be better to add "in Ethiopia" after "variation of PM2.5" in this sentence. | Thank you, we made the change | 30 |
| Line 34: It is not clear why the “six years” is mentioned here. This is not consistent with the text which shows results for 22 years. | Thank you, we made the change | 35 |
| Line 35: “Mann-Kendal” should be “Mann-Kendall”. Correct this spelling elsewhere too. | Thank you, we made the change | 36, 42, 54, 222, 224, 263 |
| Line 44: Consider changing "has existed" to "exists". | Thank you, we made the change | 45 |
| Line 48: Consider changing 'health-recommended' to 'WHO-recommended'. | Thank you, we made the change | 48 |
| Line 49: Sentence fragment. Rewrite. | Thank you, we rewritten the change | 50 |
| Line 94: “Outdoor PM2.5 level increases during winter, rainy, and spring seasons (3,4,18) than in summer and dry seasons” Is this a general statement or is it true for specific locations? Rainy seasons should see lower PM levels due to the scavenging of particulates from rain drops and cloud droplets. This should be clarified. | We accepted the comment and made explanations | 91-105 |
| Line 99 – Line 102: This statement requires citations. Here is a recent example with a focus on a South Asian country: https://doi.org/10.1016/j.atmosres.2021.105623 | Thank you, the statement is from the references cited in line 120. We made additional explanations. | 111-114 |
| Line 110: Subscript PM2.5 to be consistent with other places where it appears. | Thank you, we made the necessary change | 125, 52, 262 |
| Line 166- Line 168: This statement should cite the ACAG satellite-based PM2.5 product publication. | We accepted the comment and cited the appropriate reference | 185 |
| Line 268: Define what the high-high, low-low, high-low and low-high cluster and outliers mean. | Thank you, we have added explanations | 290-291, 293-295, 296-297, 297-299 |
| **Reviewer 2 comments** |  |  |
| **Reviewer comments** | **Authors response** | **Line number** |
| 1) The collection of lots number of Keywords, authors are requested to delete few and keep the genesis of this work. | Thank you, we removed five keywords | 53-54 |
| (2) The numbering in the introduction should be avoided in the following lines: PM can be released straight into the air or formed from emitted pollutants (1). Chemicals constituted in PM include organic carbon, elemental carbon, soil dust, sulfate, nitrate, ammonium ions (2–4), ozone (5), and heavy metals (i.e. chromium and cadmium) (6). The biological constituents (bioaerosols) of PM2.5 include allergens and microbial compounds of bacterial and fungal origins (1,7). | Thank you, we cited the references at the end of the paragraph | 81 |
| (3) Please change/modify the references in the following: “all-cause mortality (11,12,14–17)” | Thank you, we modified the references | 90 |
| (4) Rephrase the following sentences It obligates the government to regulate, monitor, and oversee compliance. However, the government lacks enough monitoring laboratories and skilled professionals to monitor air quality. As a result, evidence that influences meaningful policy actions and record-keeping regarding air pollution is undersupplied (24). To the best of our knowledge, no study in the country characterizes the geographical distribution of ambient PM2.5. This study, therefore, has assessed the spatial and temporal variation of PM2.5 in Ethiopia. | We accepted and rewrite the concept | 134-143 |
| (5) Abbreviations may be shifted into the starting of the manuscript. | Thank you, we have gone through the manuscript and looked for Abbreviations |  |
| (6) There are lots of commas and full stops are missing. | Thank you, we have gone through the manuscript and made some changes |  |
| (7) Improve fig 1 | Thank you, we have improved the graph | 259, Fig 1 |
| (8) The current study may be reflect strong impact if you can cite the following reference. -Characterization of PM10 over urban and rural sites of Rajnandgaon, central India, Natural Hazards - Review on composition, emission sources of RSPM, TSPM, heavy metals and ions with effect on environment and health, Res. J. Chem. Environ -Understanding Sources and Composition of Black Carbon and PM2.5 in Urban Environments in East India - Toxicity and health risk assessment of polycyclic aromatic hydrocarbons in surface water, sediments and groundwater vulnerability in Damodar River Basin, Groundwater for Sustainable Development 13, 100553, -Health risk assessment, composition, and distribution of polycyclic aromatic hydrocarbons (PAHs) in drinking water of Southern Jharkhand, East India, Archives of environmental contamination and toxicology 80 (1), 120-133, -Source identification and health risk assessment of atmospheric PM2. 5-bound polycyclic aromatic hydrocarbons in Jamshedpur, India, Sustainable cities and society 52, 101801 | Thank you, we cited three of these references | 81, 345 |
